# Does coinsurance reduction influence informer-sector workers’ and farmers’ utilization of outpatient care? A quasi-experimental study in China

**DOI:** 10.1186/s12913-022-08301-x

**Published:** 2022-07-14

**Authors:** Mingming Xu, Xingtong Pei

**Affiliations:** 1 School of Public Health (Shenzhen), Sun Yat-sen University, Gongchang Road 66, Shenzhen, 518107 China; 2 Department of Economics and Management, Karlsruhe Institute of Technology, Kronenstraβe 34, 76133 Karlsruhe, Germany

**Keywords:** Coinsurance reduction, Outpatient care utilization, Informal-sector workers, Farmers, China

## Abstract

**Background:**

In recent years, the Chinese government has been trying to improve informal-sector workers’ and farmers’ access to healthcare and reduce their financial burdens by introducing a plan of cost-sharing reduction, but the effect on outpatient care utilization remains unknown. Furthermore, scarce evidence has been provided to help understand the impact of cost-sharing reduction on healthcare use in low- and middle-income countries. The policy change of the coinsurance reduction for outpatient care from 75 to 55% for the enrollees of the Urban and Rural Residents Basic Medical Insurance in Taizhou, China in 2015 provides us a good quasi-experimental setting to explore such an impact.

**Methods:**

We do a quasi-experimental study to explore the impact of coinsurance reduction on outpatient care use among the informal-sector workers and farmers aged 45 and above by estimating a fixed-effects negative binomial model with the difference-in-differences approach and the matching method. Heterogeneous effects in primary care clinics and for the older people aged 60 and above are also examined. Our data is from the China Health and Retirement Longitudinal Study 2013 and 2015.

**Results:**

We find neither statistically significant impact of coinsurance reduction on outpatient care utilization in all health facilities for informal-sector workers and farmers aged 45 and above, nor heterogeneous effects in primary care clinics and for older people aged 60 and above.

**Conclusions:**

We conclude that the coinsurance reduction cannot effectively improve the informal-sector workers’ and farmers’ utilization of healthcare if the cost-sharing undertaken by patients remains high even after the reduction. Besides, improving healthcare quality in primary care clinics may play a more important role than merely introducing a cost-sharing reduction plan in enhancing the role of primary care clinics as gatekeepers. We propose that only a substantial coinsurance reduction may help influence the utilization of healthcare for informal-sector workers and farmers, and enhancing the healthcare quality in primary care clinics should be given priority in low- and middle-income countries.

**Supplementary Information:**

The online version contains supplementary material available at 10.1186/s12913-022-08301-x.

## Background

### Introduction

The cost-sharing mechanism has been utilized in health insurance systems for decades, that beneficiaries should pay a portion of costs for insurance-covered services. Its original purpose is to restrict improper healthcare utilization and reduce moral hazards [1]. In general, cost sharing adopts the following forms, deductible, copayment, coinsurance and sometimes ceiling which is set as the maximum reimbursement amount [2]. Deductible is the amount borne by patients before health insurance scheme starts to pay for the expenses. Copayment is a set amount patients need to pay before getting access to certain products or services. Coinsurance is a percentage of the total medical expenses for covered healthcare that patients have to pay out-of-pocket. Researchers have indeed demonstrated that the introduction of cost-sharing could effectively restrict healthcare utilization, however, essential utilization is also included, especially for vulnerable people [3–8].

Subsequently, to improve the vulnerable people’s access to healthcare, some high-income countries (HICs) have initiated cost-sharing reduction plans guided by the demand theory that a lower price would induce a higher demand. Some researchers from Japan found that the cost-sharing reduction could significantly improve older people’s access to healthcare [8–10]. Others from Germany saw a shorter-term increase but a longer-term reduction in access to outpatient care [11]. However, researchers from the US only detected a negligible change in access to outpatient mental healthcare [12].

Compared with HICs, informal-sector workers and farmers constitute a larger proportion of the population in low- and middle-income countries (LMICs), making LMICs might respond to cost-sharing reduction differently due to limited and unstable income for this subgroup of people. According to the available literature, some researchers have investigated the association between the New Rural Cooperative Medical Scheme (NCMS) and healthcare utilization in China and found that the introduction of the NCMS increased inpatient care utilization but not for outpatient care possibly due to the high coinsurance rates, as proposed by the authors [13, 14]. However, causal inference between the coinsurance and healthcare utilization in LMICs by adopting quasi-experimental settings still lacks among the existing evidence, partially due to the limited availability of relevant policies. Informal-sector workers and farmers tend to become vulnerable due to the following reasons. First, informal-sector workers tend to get low-skilled and low-wage jobs with worse working conditions [15]. Second, the employment status of informal-sector workers is quite unstable due to the loose labor contract between workers and employers [16]. Third, informal-sector workers and farmers have low participation in social security because of institutional constraints and employer behaviors, and in turn tend to become vulnerable when being older if without pension [17–19].

The policy change in Taizhou, China in 2015 provides us a good quasi-experimental setting to explore such an impact and fill the gap in the literature. To improve informal-sector workers’ and farmers’ access to healthcare, the government of Taizhou, Zhejiang province reduced the coinsurance, a form of cost-sharing when a fixed proportion of costs paid by enrollees, for outpatient care from 75 to 55% for the enrollees of the Urban and Rural Residents Basic Medical Insurance (URBMI) [20]. In China, informal-sector workers and farmers are mainly enrolled in URBMI according to the official policies [21, 22]. This paper intends to take advantage of this policy change and take China as an example to explore the unique impact of coinsurance reduction on outpatient care use for informal-sector workers and farmers in LMICs. We intend to explore the causal relationship between the coinsurance and healthcare utilization in LMICs by adopting a quasi-experimental setting and fill the gap in the existing literature. The findings can help understand how the coinsurance reduction affects informal-sector workers’ and farmers’ utilization of healthcare in LMICs.

### Institutional setting

The Chinese health insurance system consists of Government Medical Insurance, Urban Employee Basic Medical Insurance, and URBMI, whose enrollees are civil servants, formal employees, and informal-sector workers and farmers, respectively [23–25]. The URBMI is the combination of the original Urban Resident Basic Medical Insurance and the NCMS [26]. To maximize the population coverage of informal-sector workers and farmers, the cost coverage has to give way due to the limited insurance pool [25]. Therefore, the URBMI enrollees have to undertake higher cost-sharing (around 60-70%) for covered healthcare services than their counterparts enrolled in other insurances [25].

At the end of 2020, China achieved the goal of eliminating extreme poverty through 8 years of hard work. Among diverse factors, health as a kind of human capital is deemed as a key to poverty alleviation. According to the literature, health has a positive impact on individuals’ income and can help the poor get rid of poverty by increasing productivity [27–29]. The health shock caused by diseases will arouse the loss of human capital and in turn, reduce income [30, 31]. Therefore, China rolled out the health poverty alleviation project, committed to increasing the health insurance coverage and avoiding the catastrophic health expenses for the poor [32]. As a response to the national health poverty alleviation project, many local governments proposed comprehensive cost-sharing reduction plans to improve the poor’s access to healthcare [33, 34]. For instance, the Taizhou government gave priority to the informal-sector workers and farmers, who tend to return to poverty when the catastrophic health expenses occur.

Taizhou, a city in Zhejiang province of China initiated a coinsurance reduction plan for outpatient care utilization in primary care clinics among the enrollees of the URBMI in 2015. It regulated that the coinsurance was reduced from 75 to 55% and the ceiling was increased from 500 to 600 RMB per year [20]. To effectively allocate medical resources, the URBMI reimbursed only care use in primary care clinics. However, it is still unknown how such coinsurance reduction plans function. For instance, whether people’s access to outpatient care has been improved, and whether healthcare use has been effectively induced from secondary and tertiary health facilities to primary care clinics.

In this study, we adopt a difference-in-differences (DID) approach combined with a matching method and estimate a fixed-effects negative binomial model to explore the impact of the coinsurance reduction on outpatient care utilization in all health facilities for informal-sector workers and farmers aged 45 and above. Further, we investigate the heterogeneous impacts on outpatient care utilization in primary care clinics and for older people aged 60 and above. This study can examine the effect of the cost-sharing reduction plan in China, help understand whether it could influence informal-sector workers’ and farmers’ utilization of healthcare, and provide empirical evidence for policymakers in LMICs.

## Methods

### Data source and study population

The authors utilize data from the China Health and Retirement Longitudinal Study (CHARLS), which collects a nationally representative sample of the population aged 45 and above in China and provides individual-level panel data on health, socio-economic status, and social and family networks every 2 years [35]. The current study contains only publicly available data. Data from wave 2 (2013) and wave 4 (2015) is used in this study, corresponding to the time window of the policy change. Wave 3 is excluded since it collects life history data. Besides, economic data reflecting provincial GDP per capita is merged with the data from CHARLS [36].

The selection criteria for the sample are shown as follows. 1) Observations should be from Taizhou (treatment group) or Maoming (control group). Maoming is a city in Guangdong province, which city shares similarities in population, size, and regional GDP with Taizhou [36]. Maoming serves as the control group since no relevant policy changes between 2013 and 2015, with the coinsurance as 50%. 2) Observations should be enrolled in the URBMI (or the original Urban Resident Basic Medical Insurance and the NCMS since they were not integrated into the URBMI in Taizhou in 2013). Informal-sector workers and farmers are the target observations in this study. We include enrollees in the URBMI because they are informal-sector workers, farmers or children (which does not apply to our study since our sample are aged 45 and above) according to the policy documents [37]. 3) Individuals should be surveyed in both 2013 and 2015 since we need to detect the variation within the group. 4) Only non-missing data for doctor visits are included. 5) Observations should be aged 45 and above. In the end, 664 observations are included in the study, among which 298 are in the treatment group.

Descriptive statistics are presented in Table 1. It indicates that the average number of doctor visits was unbalanced between the treatment and control groups. To avoid selection bias, we matched the two groups according to relevant pre-treatment variables, as shown in the section of Design and statistical analysis.

**Table 1:** Descriptive statistics of 664 observations in 2013 and 2015

	2013		2015	
	Taizhou (treatment)	Maoming (control)	Taizhou (treatment)	Maoming (control)
Doctor visits, Mean (SD)				
All health facilities	0.21 (0.84)	0.86 (1.91)	0.23 (0.93)	0.71 (1.54)
Secondary & tertiary hospitals	0.11 (0.46)	0.12 (0.44)	0.15 (0.79)	0.066 (0.34)
Primary care clinics	0.097 (0.72)	0.61 (1.75)	0.081 (0.50)	0.61 (1.53)
Equivalent income, RMB				
Quartile (25%)	678.8	370.9	0	0
Quartile (50%)	7205.3	4527.1	6400	2206.2
Quartile (75%)	26,474.8	18,073.8	26,634.1	17,150
Marital status, %				
The married	84.6	86.3	84.6	85.2
The single	15.4	13.7	15.4	14.8
No. of chronic diseases, Mean (SD)	0.88 (1.06)	0.76 (1.09)	1.05 (1.13)	0.91 (1.07)
Self-perceived health status, %				
Excellent	12.1	2.75	7.69	2.89
Very good	22.1	8.24	11.9	8.67
Good	24.2	35.2	34.3	28.9
Fair	28.9	37.9	37.1	43.9
Poor	12.8	15.9	9.09	15.6
Age, Mean (SD)	61.8 (10.1)	60.4 (10.1)	63.6 (10.0)	62.1 (10.0)
Gender, %				
Male	47.7	45.4	47.7	45.4
Female	52.3	54.6	52.3	54.6
Education attainment, %				
No education	36.2	35.2	35.6	31.1
Elementary, middle school	58.4	54.9	49.0	37.2
High school and above	5.37	9.89	15.4	31.7
Occupation, %				
Agricultural work	34.8	46.4	28.8	52.4
Employed	25.4	17.1	24.5	18.5
Self-employed	19.6	12.2	16.5	6.55
Retired/receded	0.72	2.21	0.72	1.19
Unemployed	19.6	22.1	29.5	21.4
Household number, Mean (SD)	4.14 (1.72)	5.53 (2.61)	2.74 (1.44)	3.46 (1.97)
Provincial GDP per capita (RMB)	68,036	58,860	78,768	69,283
n	149	183	149	183

Notes. SD Standard deviation (in parentheses)

### Measures and variables

The dependent variable is the number of doctor visits in the last 4 weeks. We first look at the doctor visits in all health facilities and investigate the aggregated impact on outpatient care use. We then focus on the doctor visits in primary care clinics since the coinsurance reduction policy concerns only primary care clinics.

Consistent with the relevant literature [38], we control for the following time-variant covariates: quartiles of equivalent income (household income divided by the square root of household size) [39]; marital status (1 = married with spouse present, married but not living with spouse temporarily for reasons such as work, and cohabitated, for short, [married], 2 = separated, divorced, widowed and never married, for short, [single]); the number of chronic diseases; self-perceived health status (1 = excellent, 2 = very good, 3 = good, 4 = fair, 5 = poor); education attainment (1 = no education, 2 = elementary/middle school, 3 = high school and above); occupation (1 = agricultural work, 2 = employed, 3 = self-employed, 4 = retired/receded, 5 = unemployed); household number and provincial GDP per capita.

### Design and statistical analysis

We explore the impact of coinsurance reduction on outpatient care use by estimating a fixed-effects negative binomial model with the DID approach and the matching method. Combining the DID and matching could lower the bias compared with a single method of regression or matching according to the literature [40, 41]. Details are shown below.

#### Matching method

We adopt a propensity score matching (PSM) method to balance the treatment and control groups and minimize selection bias. The following pre-treatment variables are considered in estimating the Probit model: 1) time-invariant variables, such as the birth year and gender; 2) time-variant variables, such as the number of doctor visits, marital status, equivalent income, the number of chronic diseases, self-perceived health status, education, occupation, and household size. The kernel algorithm is used to give a larger weight on observations with smaller distance metrics [42].

We assess the matching quality according to match rates, standardized differences, and propensity score density. First, the match rates for the two groups were approximately 95% and most of the information in the original sample is used in the final regression (see Additional file 1: Appendix 1 for more details). Second, we calculate the standardized mean differences by dividing the mean differences by the average standard deviation of the two groups [43]. Results show that the standardized mean differences moved closer towards zero after matching and were at around 0.1, an acceptable level according to the literature [43]. This indicates the treatment and control groups were well balanced after the PSM procedure (see Additional file 2: Appendix 2 for more details). Third, the propensity score density for the two groups before and after matching indicates that the two groups were very well balanced (see Additional file 3: Appendix 3 for more details).

#### DID approach

To investigate the causal relationship between the coinsurance reduction and outpatient care use, we adopt a DID approach after conducting the PSM. According to our data, 99.72% of the observations were collected after July 2015 [44], while the coinsurance reduction plan was initiated in January 2015, enabling us to detect the policy effect by adopting the DID design [20]. With this approach, we can explore the causal relationship when the conditional independence assumption (CIA) and the identical trend assumption are met [45, 46]. First, under the CIA, variables that affect both the treatment assignment and the outcome variables should be observable. And the confounding caused by the dependence between the treatment assignment and outcome variables could be removed when these observable variables are controlled for. In this study, we control for time-invariant confounders by adding individual fixed-effects in the model, and we control for the remaining time-variant confounders by adding the observable covariates in the model. Second, we do the matching before estimating our model to help satisfy the identical trend assumption, though we cannot test this assumption with limited waves before the treatment.

#### Fixed-effects negative binomial model

Since our dependent variable is count data and there exists an overdispersion issue, we utilize a fixed-effects negative binomial model in this study [47]. Our econometric model is shown as follow:

$${y}_{it}=\exp \left(\alpha {Treat}_i+\beta {Post}_t+\gamma \left({Treat}_i\ast {Post}_t\right)+{Year}_t+{X}_{it}+{\nu}_i+{\mu}_{it}\right)$$

In this model, *y* denotes the number of doctor visits, *Treat* (1 = treatment group, 0 = control group) and *Post* (1 = after the policy change, 0 = before the policy change) are treatment and time dummy, respectively. Year and *ν* control for the time and individual (all time-invariant variables, such as birth year and gender) fixed effects, respectively. *X* denotes all the covariates. *μ* is the idiosyncratic error term, and *γ* shows the estimated treatment effect.

## Results

Table 2 lists the impact of coinsurance reduction on outpatient care utilization by presenting the incidence rate ratios (IRRs). Column [1] shows the impact on doctor visits in all health facilities for informal-sector workers and farmers aged 45 and above. The DID estimate shows that the number of doctor visits in all health facilities after the coinsurance reduction was approximately 70% of the number before the reduction. However, this is not statistically significant, indicating that a 20% reduction in coinsurance could not arouse a significant change in outpatient care utilization in the setting of Taizhou.

**Table 2:** The impact of coinsurance reduction on outpatient care utilization

	Incidence rate ratios of doctor visits		
	All health facilities		Primary care clinics
	(1) Age > =45	(2) Age > =60	(3) Age > =45
Treatment * year	0.697 (0.392)	0.874 (1.003)	0.035 (11.00)
Year, 1 (2013, ref)			
Year, 2 (2015)	0.923 (0.358)	1.493 (0.869)	5.003 (808.2)
Equivalent income: quartile 1 (ref)			
Equivalent income: quartile 2	0.213^**^(0.131)	0.957 (1.374)	0.696 (0.648)
Equivalent income: quartile 3	0.313^*^(0.198)	0.0561^**^(0.080)	0.331 (0.433)
Equivalent income: quartile 4	0.191^**^(0.137)	0.0240^**^(0.041)	0.574 (0.710)
Marital status: the married (ref)			
Marital status: the single	0.204 (0.352)	0.0331 (0.106)	0.0446 (0.246)
No. of chronic diseases	1.439 (0.435)	2.187^*^(1.010)	2.006 (1.073)
Health status (ref: Excellent)			
Very good	0.0911 (0.154)	0.000 (0.000)	0.361 (1.225)
Good	0.0640^*^(0.102)	0.000 (0.001)	0.0986 (0.257)
Fair	0.0575^*^(0.0885)	0.000 (0.000)	0.0827 (0.216)
Poor	0.0696^*^(0.110)	0.000 (0.000)	0.203 (0.558)
Education attainment (ref: No education)			
Elementary, middle school	1.038 (1.076)	1.482 (5.154)	3.046 (7.754)
High school and above	2.453 (3.330)	27.83 (105.2)	4.022 (11.22)
Occupation (ref: Agricultural work)			
Employed	3.611 (3.697)	4.355 (12.91)	2.724 (4.917)
Self-employed	0.168^*^(0.180)	0.236 (0.935)	0.008 (0.0244)
Retired/receded	–	–	–
Unemployed	0.253^*^(0.204)	0.002^**^(0.006)	0.001^*^(0.005)
Provincial GDP per capita	0.864 (0.203)	0.818 (0.396)	0.040 (16.23)
Household number	0.988 (0.123)	1.263 (0.252)	1.078 (0.172)
n	664	360	664

Notes. Estimates stem from conditional fixed-effects negative binomial specifications. Coefficients represent incidence rate ratios. Standard errors are in parentheses. Significance levels: ***p < 0.01; **p < 0.05; *p < 0.1

In column [2], we further investigate the impact of coinsurance reduction on outpatient care utilization among the older people aged 60 and above, since the older people are demonstrated to be sensitive to price changes according to the literature [8]. However, we still find no significant impacts. It seems a 20% reduction in coinsurance will not make a significant difference, even for older people.

In column [3], we try to explore whether the coinsurance reduction has an impact on doctor visits in primary care clinics since the coinsurance reduction policy concerns only care use in primary care clinics. However, no significant findings are achieved. The DID estimate demonstrates that the coinsurance reduction in Taizhou did not arouse a change of doctor visits in primary care clinics.

## Discussion

Insignificant results may indicate significant implications. In this study, we found that a 20% reduction of coinsurance cannot significantly improve the informal-sector workers’ and farmers’ utilization of outpatient care if the coinsurance remains high even after the reduction (55%). A 55% coinsurance is indeed an unneglectable financial burden for middle-aged and older people, especially for the informal-sector workers or farmers without pension. Too much coinsurance may hinder these people to see a doctor even with care needs and limited reduction cannot trigger a proper change in care use [25]. This is consistent with some prior studies during the NCMS period. Some researchers propose that the high coinsurance rates may hinder both the outpatient care and inpatient care use, especially for the low-income individuals [13, 14]. A study done in the US shows that a $17 reduction of copayment had little impact on mental healthcare utilization among the older people [12]. Only if the coinsurance has been reduced to a proper level, people’s utilization of healthcare can be improved. Some researchers in Japan indicate that the coinsurance reduction from 30 to 10% could significantly improve older people’s access to healthcare [8, 9]. This may partially explain why the enrollees of the URBMI were not significantly impacted by the coinsurance reduction policy.

Furthermore, the results show that a 20% reduction of coinsurance cannot attract patients from secondary and tertiary hospitals to primary care clinics. The lagged quality improvement in primary care clinics in China still pushes patients to secondary and tertiary health facilities, no matter for major diseases or minor illnesses, which leads to a waste of high-tech medical resources [48]. To tackle this issue, many local governments regulate that only care use in primary care clinics could be covered by the URBMI, whose purpose is to induce patients to first contact doctors in primary care clinics and make primary care clinics function as gatekeepers [20]. However, according to our findings, such a purpose seems not to be accomplished. Focusing on improving healthcare quality in primary care clinics may be a better choice.

The above findings may have the following policy implications. First, only a substantial coinsurance reduction may help improve the utilization of healthcare for informal-sector workers and farmers in LMICs. In LMICs, the cost-sharing undertaken by patients is usually high due to limited funding pools of health insurance [49, 50]. Policymakers should consider a substantial cost-sharing reduction plan to trigger an effective impact on people’s healthcare-seeking behaviors. Second, enhancing the healthcare quality in primary care clinics should be given priority in LMICs. Merely increasing the reimbursement rate, i.e., decreasing the cost-sharing cannot help primary care clinics function as gatekeepers. Enhancing the healthcare quality should be simultaneously conducted, which is also a fundamental obstacle to improving people’s access to healthcare in primary health facilities. Third, other health reforms such as Diagnosis Related Groups (DRGs) / Big Data Diagnosis-Intervention Packet (DIP) payment methods should be simultaneously introduced and adopted by hospitals. Limited coinsurance reduction may not trigger a change in healthcare utilization among the informal-sector workers and farmers, while DRGs/DIP reforms possibly help make it. Since 2019, the Chinese National Healthcare Security Administration has introduced the DRGs/DIP reforms in pilot regions [51, 52], whose purpose is to prevent the moral hazard from the doctors and improve the efficiency of health insurance fund, which would finally result in the improved coverage (either covering more healthcare services or less coinsurance) of the enrollees. This may in turn improve the access of the informal-sector workers and farmers and influence the utilization of healthcare.

There are some limitations to this study. First, we achieved no statistically significant findings possibly due to the limited sample size. However, we cannot expand our sample size by including more observations from different cities since health insurance policies vary substantially among cities. Second, we could not examine the hospitalization offset effect, i.e., whether the coinsurance reduction for outpatient care has an impact on inpatient care utilization because coinsurance policies for inpatient care also changed between 2013 and 2015.

## Conclusions

This study examines the effect of cost-sharing reduction in LMICs by taking China as an example. We conclude that such a policy measure cannot effectively improve the informal-sector workers’ and farmers’ utilization of healthcare if the cost-sharing undertaken by patients remains high even after the reduction. Besides, improving healthcare quality in primary care clinics may play a more important role than merely introducing a cost-sharing reduction plan in enhancing the role of primary care clinics as gatekeepers. Our findings could help understand how the cost-sharing reduction affects informal-sector workers’ and farmers’ access to healthcare in LMICs, fill the relevant gaps in the available literature, and provide empirical evidence for policymakers in LMICs.

## Supplementary Information

**Additional file 1: Appendix 1.** The match rates for the treatment and control groups.

**Additional file 2: Appendix 2.** The standardized mean differences between the treatment and control groups before and after matching (above: Figure; below: Table).

**Additional file 3: Appendix 3. Figure.** Propensity score density for the treatment and control groups before and after matching.

## Data Availability

The datasets generated and/or analyzed during the current study are available in the China Health and Retirement Longitudinal Study (CHARLS) repository, [http://charls.pku.edu.cn/en/].

## Abbreviations

HICs: High-income countries
LMICs: Low- and middle-income countries
URBMI: Urban and Rural Residents Basic Medical Insurance
DID: Difference-in-differences
CHARLS: China Health and Retirement Longitudinal Study
PSM: Propensity score matching
CIA: Conditional independence assumption
IRRs: Incidence rate ratios
