# Supplementary material for: Does coinsurance reduction influence informer-sector workers’ and farmers’ utilization of outpatient care? A quasi-experimental study in China
Source: BMC Health Serv Res. 2022 Jul 14;22:914. doi: 10.1186/s12913-022-08301-x (PMC9281155; doi:10.1186/s12913-022-08301-x)
Supplement: Supplementary file 1 — Additional file 1: Appendix 1. The match rates for the treatment and control groups. [file 12913_2022_8301_MOESM1_ESM.docx]

**Appendix 1.**

**The match rates for the treatment and control groups**

|  | Matched | Total | **Match rates** |
| --- | --- | --- | --- |
| Treatment | 126 | 129 | **97.7%** |
| Control | 164 | 170 | **96.5%** |
| Combined | 290 | 299 | **97.0%** |
