# Supplementary material for: Does coinsurance reduction influence informer-sector workers’ and farmers’ utilization of outpatient care? A quasi-experimental study in China
Source: BMC Health Serv Res. 2022 Jul 14;22:914. doi: 10.1186/s12913-022-08301-x (PMC9281155; doi:10.1186/s12913-022-08301-x)
Supplement: Supplementary file 2 — Additional file 2: Appendix 2. The standardized mean differences between the treatment and control groups before and after matching (above: Figure; below: Table). [file 12913_2022_8301_MOESM2_ESM.docx]

**Appendix 2.**

The standardized mean differences between the treatment and control groups before and after matching (above: Figure; below: Table)

|  | **Before matching** | | | **After matching** | | |
| --- | --- | --- | --- | --- | --- | --- |
|  | Treatment | Control | SD | Treatment | Control | SD |
| Doctor visits | 0.22 | 0.87 | -0.43 | 0.55 | 0.53 | 0.01 |
| Marital status | 0.16 | 0.12 | 0.11 | 0.12 | 0.14 | -0.05 |
| Equivalent income | 14124 | 10451 | 0.21 | 11428 | 12450 | -0.06 |
| No. of chronic diseases | 0.87 | 0.76 | 0.10 | 0.80 | 0.87 | -0.06 |
| Health status (ref: Excellent) |  |  |  |  |  |  |
| Very good | 0.22 | 0.08 | 0.40 | 0.15 | 0.15 | 0.00 |
| Good | 0.23 | 0.36 | -0.28 | 0.33 | 0.31 | 0.03 |
| Fair | 0.29 | 0.37 | -0.18 | 0.31 | 0.32 | -0.02 |
| Poor | 0.12 | 0.16 | -0.10 | 0.15 | 0.16 | -0.04 |
| Gender | 0.51 | 0.54 | -0.06 | 0.46 | 0.53 | -0.15 |
| Age | 61.71 | 60.11 | 0.16 | 60.61 | 61.18 | -0.06 |
| Education attainment (ref: No education) |  |  |  |  |  |  |
| Elementary, middle school | 0.62 | 0.56 | 0.11 | 0.60 | 0.57 | 0.07 |
| High school and above | 0.05 | 0.11 | -0.22 | 0.07 | 0.08 | -0.03 |
| Occupation (ref: Agricultural work) |  |  |  |  |  |  |
| Employed | 0.26 | 0.16 | 0.24 | 0.21 | 0.24 | -0.07 |
| Self-employed | 0.21 | 0.13 | 0.21 | 0.17 | 0.16 | 0.03 |
| Retired/receded | 0.01 | 0.02 | -0.13 | 0.01 | 0.01 | 0.03 |
| Unemployed | 0.20 | 0.22 | -0.04 | 0.25 | 0.22 | 0.06 |
| Household size | 3.97 | 5.36 | -0.65 | 4.57 | 4.70 | -0.06 |

Note. SD=Standardized difference.
