# Supplementary material for: Does coinsurance reduction influence informer-sector workers’ and farmers’ utilization of outpatient care? A quasi-experimental study in China
Source: BMC Health Serv Res. 2022 Jul 14;22:914. doi: 10.1186/s12913-022-08301-x (PMC9281155; doi:10.1186/s12913-022-08301-x)
Supplement: Supplementary file 3 — Additional file 3: Appendix 3. Figure. Propensity score density for the treatment and control groups before and after matching. [file 12913_2022_8301_MOESM3_ESM.docx]

**Appendix 3.**

**Figure.** Propensity score density for the treatment and control groups before and after matching
